# Reduced Fatty Acid Use from CD36 Deficiency Deteriorates Streptozotocin-Induced Diabetic Cardiomyopathy in Mice

**DOI:** 10.3390/metabo11120881

**Published:** 2021-12-17

**Authors:** Yogi Umbarawan, Ryo Kawakami, Mas Rizky A. A. Syamsunarno, Hideru Obinata, Aiko Yamaguchi, Hirofumi Hanaoka, Takako Hishiki, Noriyo Hayakawa, Norimichi Koitabashi, Hiroaki Sunaga, Hiroki Matsui, Masahiko Kurabayashi, Tatsuya Iso

**Affiliations:** 1 Department of Cardiovascular Medicine, Gunma University Graduate School of Medicine, 3-39-22 Showa-Machi, Maebashi 371-8511, Japan; umbarawans@yahoo.com (Y.U.); r.kawakami.1526@gmail.com (R.K.); masrizkyanggun@yahoo.com (M.R.A.A.S.); norikoitabashi@gmail.com (N.K.); hsunaga@gunma-u.ac.jp (H.S.); mkuraba@gunma-u.ac.jp (M.K.); 2 Department of Internal Medicine, Faculty of Medicine Universitas Indonesia, Jl. Salemba Raya no. 6, Jakarta 10430, Indonesia; 3 Department of Biomedical Sciences, Universitas Padjadjaran, Jl. Raya Bandung Sumedang KM 21, Jatinangor 45363, Indonesia; 4 Education and Research Support Center, Gunma University Graduate School of Medicine, 3-39-22 Showa-Machi, Maebashi 371-8511, Japan; obi@gunma-u.ac.jp; 5 Department of Bioimaging Information Analysis, Gunma University Graduate School of Medicine, 3-39-22 Showa-Machi, Maebashi 371-8511, Japan; Aiko.Yamaguchi@uth.tmc.edu (A.Y.); hanaokah@gunma-u.ac.jp (H.H.); 6 Department of Biochemistry, Keio University School of Medicine, 35 Shinano-Machi, Shinjuku-Ku, Tokyo 160-8582, Japan; htakako@keio.jp (T.H.); ha_noriyo@z5.keio.jp (N.H.); 7 Clinical and Translational Research Center, Keio University School of Medicine, 35 Shinano-Machi, Shinjuku-Ku, Tokyo 160-8582, Japan; 8 Center for Liberal Arts and Sciences, Ashikaga University, 268-1 Omae-Machi, Ashikaga 326-8558, Japan; 9 Department of Laboratory Sciences, Gunma University Graduate School of Health Sciences, 3-39-22 Showa-Machi, Maebashi 371-8511, Japan; hmatsui@gunma-u.ac.jp; 10 Department of Medical Technology and Clinical Engineering, Gunma University of Health and Welfare, 191-1 Kawamagari-Machi, Maebashi 371-0823, Japan

**Keywords:** diabetic cardiomyopathy, streptozotocin, CD36, glucose, fatty acid, ceramide, metabolomics

## Abstract

Cardiac dysfunction is induced by multifactorial mechanisms in diabetes. Deranged fatty acid (FA) utilization, known as lipotoxicity, has long been postulated as one of the upstream events in the development of diabetic cardiomyopathy. CD36, a transmembrane glycoprotein, plays a major role in FA uptake in the heart. CD36 knockout (CD36KO) hearts exhibit reduced rates of FA transport with marked enhancement of glucose use. In this study, we explore whether reduced FA use by CD36 ablation suppresses the development of streptozotocin (STZ)-induced diabetic cardiomyopathy. We found that cardiac contractile dysfunction had deteriorated 16 weeks after STZ treatment in CD36KO mice. Although accelerated glucose uptake was not reduced in CD36KO-STZ hearts, the total energy supply, estimated by the pool size in the TCA cycle, was significantly reduced. The isotopomer analysis with ^13^C_6_-glucose revealed that accelerated glycolysis, estimated by enrichment of ^13^C_2_-citrate and ^13^C_2_-malate, was markedly suppressed in CD36KO-STZ hearts. Levels of ceramides, which are cardiotoxic lipids, were not elevated in CD36KO-STZ hearts compared to wild-type-STZ ones. Furthermore, increased energy demand by transverse aortic constriction resulted in synergistic exacerbation of contractile dysfunction in CD36KO-STZ mice. These findings suggest that CD36KO-STZ hearts are energetically compromised by reduced FA use and suppressed glycolysis; therefore, the limitation of FA utilization is detrimental to cardiac energetics in this model of diabetic cardiomyopathy.

## 1. Introduction

Diabetes is an independent risk factor for the development of heart failure. Although diabetic cardiomyopathy is likely to be induced by multifactorial mechanisms, lipotoxicity is suggested to play a significant role [1,2,3,4,5]. Deranged FA utilization in the heart has been shown to occur in diabetes in humans and animals. FA uptake and oxidation are presumably accelerated to compensate for decreased glucose use. In cardiomyocytes, excessive FA is converted into lipid intermediates such as triacylglycerol (TG) and others with lipotoxic effects (e.g., diacylglycerol and ceramides). Increased ceramide levels can activate inflammatory signaling pathways and increase the production of reactive oxygen species, which, in turn, results in impairment of mitochondrial energy production. Reduced cardiac efficiency (cardiac work/oxygen consumption) by deranged FA use is also suggested to be causative of diabetic cardiomyopathy. To date, the lipotoxicity hypothesis prevails as one of most upstream events to trigger cardiac dysfunction in diabetes [1,2,3,4,5].

CD36, also referred to as fatty acid translocase (FAT), is a single-chain 88 kDa glycoprotein that has wide biological functions in various kinds of cells, such as adipocytes, macrophages, the endothelium, skeletal muscle and cardiomyocytes [1,6]. In the heart, CD36 is more abundant in the capillary endothelium than in cardiomyocytes and plays a significant role in FA uptake and oxidation [7,8]. Whole CD36-knockout (CD36KO) hearts exhibit reduced rates of FA transport and oxidation with marked enhancement of glucose use [7,9,10]. Endothelial-specific CD36KO mice recapitulate the metabolic phenotype of whole CD36KO hearts, while cardiac-specific CD36KO hearts exhibit only a minor alternation in metabolism [7,9,10]. In the context of metabolic diseases such as diabetes and hyperlipidemia, CD36 has been suggested to be a key molecule to affect cardiac function [1,6,11]. The expression levels of CD36 are upregulated with its translocation to the sarcolemma in diabetes and hyperlipidemia. An increase in myocardial lipid transport and accumulation is suppressed by CD36 deficiency in mice, which is thought to be beneficial for lipid-mediated cardiac dysfunction models, such as age-induced cardiomyopathy [12] and transgenic mice overexpressing PPARα in the myocardium [13]. Despite the beneficial impact of CD36 ablation on several metabolic stresses, direct effects of CD36 deletion on cardiac function in diabetes have not been explored in vivo.

In this study, we addressed the question of whether limited FA use, as a result of CD36 deletion, is protective against the development of diabetic cardiomyopathy generated by streptozotocin (STZ) treatment. In contradiction to our assumption, we found that cardiac contractile dysfunction was exacerbated in STZ-treated CD36KO mice. Nuclear medicine and metabolomics analyses revealed that the total energy supply was diminished in CD36KO-STZ hearts due to a reduction in FA uptake and suppressed glycolysis. An increased workload by transverse aortic constriction (TAC) further aggravated the cardiac contractile dysfunction. Our data suggest that CD36KO-STZ hearts are energetically compromised by reduced FA use and suppressed glycolysis. Thus, it is plausible that enhanced FA utilization is favorable for diabetic hearts where glucose use is impaired because of insulin depletion and that limited FA use could deteriorate cardiac energetics in this type of diabetic cardiomyopathy.

## 2. Results

### 2.1. Pronounced Left Ventricular Contractile Dysfunction in STZ-Treated CD36KO Mice

**Figure 1:**
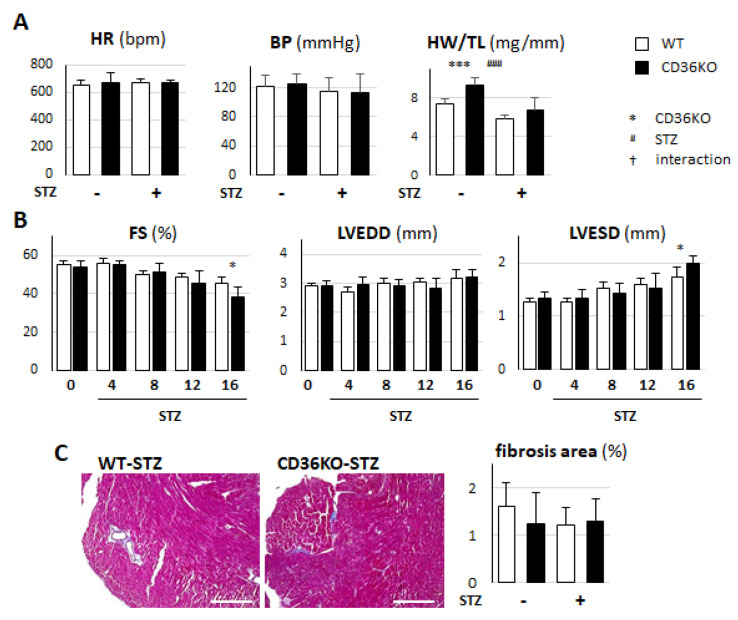
Cardiac contractile dysfunction was more greatly deteriorated in CD36KO-STZ mice. (**A**) Heart rate (HR) and systolic blood pressure (BP) were not significantly different between the two groups. Heart weight/tibial length (HW/TL) ratio was significantly reduced 16 weeks after STZ treatment. (**B**) Cardiac function was estimated by echocardiography before STZ treatment and 4, 8, 12 and 16 weeks after (*n* = 10–12). FS, fractional shortening; LVEDD, left ventricular end-diastolic diameter; LVESD, left ventricular end-systolic diameter. (**C**) Cardiac fibrosis was estimated by Masson’s trichrome stain (*n* = 6). Scale bar = 200 µm. * *p* < 0.05, *** *p* < 0.001; main effect for genotype. ^###^ *p* < 0.001; main effect for STZ treatment.

To study whether reduced FA use is protective against diabetic cardiomyopathy, wild-type (WT) and CD36-knockout (CD36KO) mice were treated with STZ and observed for 16 weeks. The heart rate and systolic blood pressure were not significantly different between the groups with and without STZ treatment (Figure 1A). Although the heart weight relative to the tibial length was higher in CD36KO mice at the baseline, cardiac atrophy was similarly induced in both STZ-treated groups (Figure 1A). The echocardiographic analysis revealed that, while no functional difference between WT and CD36KO mice was observed at the baseline, a decrease in fractional shortening (FS) and an increase in left ventricular end-systolic diameter (LVESD) were more pronounced in CD36KO-STZ mice at 16 weeks (Figure 1B). The fibrosis area was not altered by STZ treatment and was comparable between the two groups (Figure 1C). Thus, CD36KO-STZ mice exhibited more prominent contractile dysfunction compared to WT-STZ mice with similar cardiac atrophy and no obvious fibrosis 16 weeks after the onset of diabetes.

### 2.2. Accelerated Glucose Uptake and Low Fatty Acid Uptake in CD36KO-STZ Hearts

**Figure 2:**
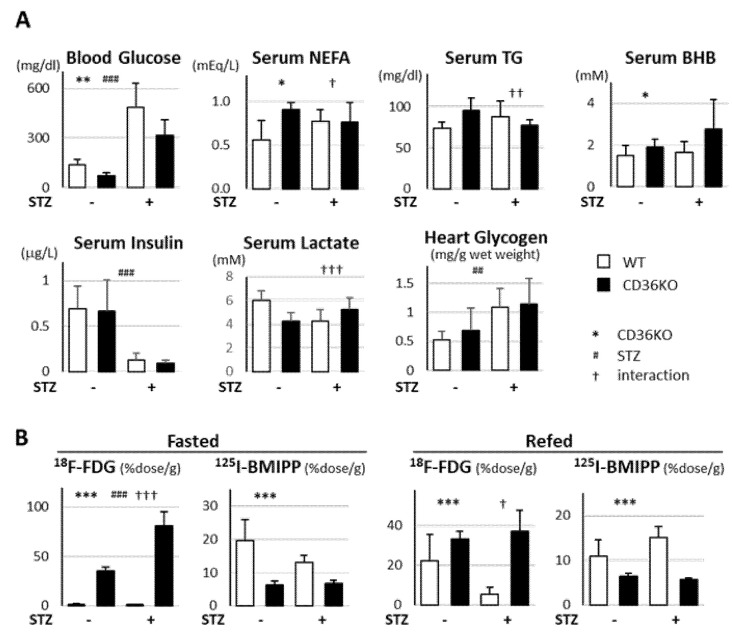
Circulating levels of metabolites and uptake of glucose and FA tracers by hearts in absence or presence of STZ treatment. (**A**) Blood samples were collected after a 6 h fast to measure serum levels of glucose, NEFA, TG, BHB, insulin and lactate (*n* = 5–7). Glycogen in hearts was also measured. NEFA, non-esterified fatty acids; TG, triacylglycerol; BHB, β-hydroxybutyrate. (**B**) Uptake of ^18^F-FDG (glucose tracer) and ^125^I-BMIPP (FA tracer) by hearts was assessed 2 h after intravenous injection (*n* = 5–7). * *p* < 0.05, ** *p* < 0.01, *** *p* < 0.001; main effect for genotype. ^#^ *p* < 0.05, ^##^ *p* < 0.01, ^###^ *p* < 0.001; main effect for STZ treatment. ^†^ *p* < 0.05, ^††^ *p* < 0.01, ^†††^ *p* < 0.001; interaction between genotype and STZ treatment.

Next, we measured the metabolic parameters 16 weeks after STZ treatment (Figure 2A). The blood glucose levels were lower in CD36KO mice at the baseline and were markedly increased in both mice by STZ treatment. The serum levels of insulin were significantly reduced by STZ treatment. The serum levels of non-esterified FA (NEFA) were higher in CD36KO mice at the baseline, but comparable after STZ treatment. The serum levels of β-hydroxybutyrate (BHB) were higher in CD36KO mice. Next, we examined the cardiac uptake of 18F-FDG (glucose tracer) and 125I-BMIPP (fatty acid tracer; Figure 2B). 18F-FDG uptake was 30-fold higher in CD36KO hearts than WT at the baseline to compensate for reduced FA uptake. 18F-FDG uptake was more enhanced by STZ treatment in CD36KO hearts in the fasted state. However, 18F-FDG uptake was markedly reduced after refeeding in WT-STZ hearts, but not in CD36KO-STZ ones. These findings suggest that insulin-dependent glucose uptake is significantly reduced during the postprandial period in WT-STZ hearts, while glucose uptake is markedly enhanced independently of insulin in CD36KO. Glycogen levels in hearts were elevated by STZ treatment even in WT mice (Figure 2A), suggesting that glycogenolysis is suppressed by STZ treatment. 125I-BMIPP uptake was significantly reduced in CD36KO hearts irrespective of the feeding conditions and STZ treatment. Although 125I-BMIPP uptake tended to be reduced in WT-STZ hearts in the fasted state, 125I-BMIPP uptake was prone to being elevated after refeeding. These findings suggest that WT-STZ hearts depend more on FA as fuel, while CD36KO-STZ hearts utilize more glucose.

### 2.3. STZ Treatment Suppresses Enhanced Glycolytic Rate in CD36KO Hearts

**Figure 3:**
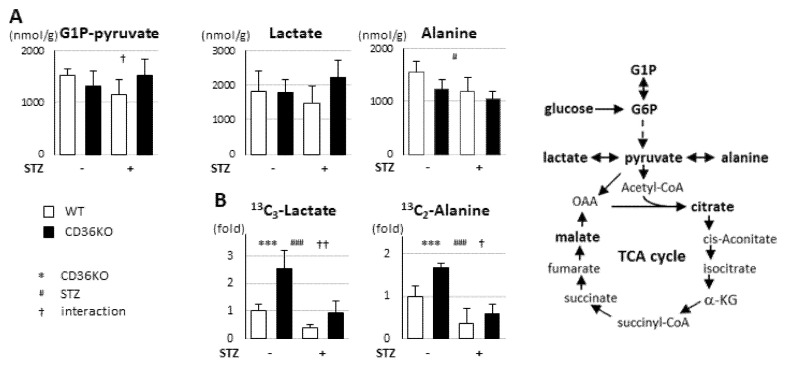
Glycolytic flux was markedly suppressed by STZ treatment. Hearts were isolated after a 6 h fast for metabolome analysis. (**A**) Metabolic profiling in glycolysis pathway. G1P-pyruvate, total metabolites from G1P to pyruvate in glycolysis (*n* = 5–7). (**B**) Tracer study with ^13^C_6_-glucose. After a 6 h fast, hearts were isolated 10 min after intraperitoneal injection of ^13^C_6_-glucose (*n* = 5–7). G1P, glucose 1-phosphate; G6P, glucose 6-phosphate; α-KG, α-ketoglutarate; OAA, oxaloacetate. *** *p* < 0.001; main effect for genotype. ^#^ *p* < 0.05, ^###^ *p* < 0.001; main effect for STZ treatment. ^†^ *p* < 0.05, ^††^ *p* < 0.01; interaction between genotype and STZ treatment.

To estimate the metabolic alterations in CD36KO-STZ hearts, we performed metabolome analysis. Despite a dramatic increase in glucose uptake in CD36KO hearts (Figure 2B), the total metabolites in glycolysis (G1P to pyruvate) were comparable between WT and CD36KO hearts at the baseline (Figure 3A). The levels of G1P to pyruvate trended to be reduced in WT-STZ hearts, while they were prone to being elevated in CD36-STZ (Figure 3A; interaction, *p* < 0.05). The levels of lactate were not different, while the levels of alanine were reduced by STZ treatment (Figure 3A). However, a tracer study with 13C6-glucose revealed that the levels of 13C3-lactate and 13C2-alanine were significantly increased in CD36KO hearts compared to WT at the baseline, but the flux was more greatly suppressed by STZ treatment in CD36KO hearts (Figure 3B; interaction, *p* < 0.01 for 13C3-lactate and *p* < 0.05 for 13C2-alanine). These data suggest that the accelerated glycolytic rate in CD36KO hearts is strongly suppressed by insulin deficiency, despite a sustained increase in glucose uptake.

### 2.4. Reduced FA Uptake and Suppressed Glycolysis Diminish Total Energy Supply in CD36KO-STZ Hearts

**Figure 4:**
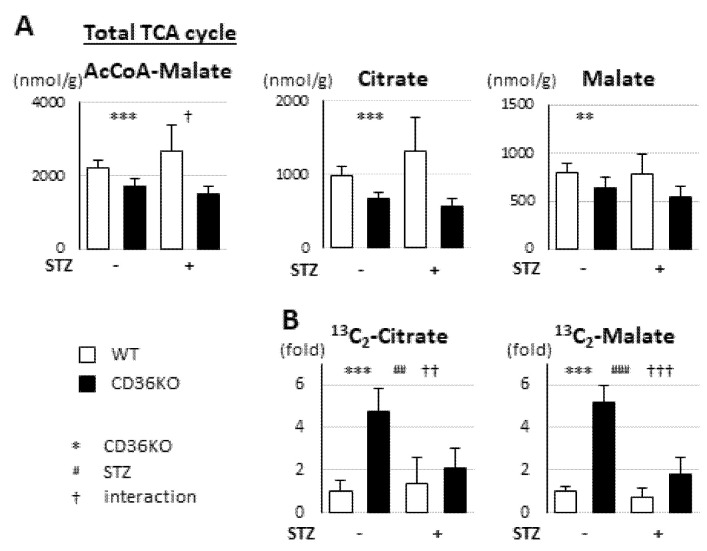
Pool size in TCA cycle was significantly reduced in CD36KO-STZ hearts. Hearts were isolated after a 6 h fast for metabolome analysis. (**A**) Metabolic profiling in TCA cycle (*n* = 5–7). TCA cycle, tricarboxylic acid cycle. (**B**) Tracer study with ^13^C_6_-glucose. After a 6 h fast, hearts were isolated 10 min after intraperitoneal injection of ^13^C_6_-glucose (*n* = 5–7). ** *p* < 0.01, *** *p* < 0.001; main effect for genotype. ^##^ *p* < 0.01, ^###^ *p* < 0.001; main effect for STZ treatment. ^†^ *p* < 0.05, ^††^ *p* < 0.01, ^†††^ *p* < 0.001; interaction between genotype and STZ treatment.

We next studied the metabolic changes in the TCA cycle. The pool size of total metabolites in the TCA cycle (acetyl-CoA to malate) was significantly reduced in CD36KO hearts and the difference was enhanced by STZ treatment (Figure 4A; interaction, *p* < 0.05). Consistent with this, each metabolite in the TCA cycle, such as citrate and malate, was lower in CD36KO mice and the difference tended to be enhanced (Figure 4A). The isotopomer analysis with 13C6-glucose revealed that the levels of 13C2-citrate and 13C2-malate were markedly elevated in CD36KO hearts at the baseline, while they were strongly suppressed by STZ treatment, despite sustained enhancement of glucose uptake (Figure 4B; interaction, *p* < 0.01 for 13C2-citrate, *p* < 0.001 for 13C2-malate). Taken together, we surmise that the total energy supply, estimated by the pool size in the TCA cycle, is markedly diminished by reduced FA uptake and suppressed glycolysis in CD36KO hearts, which can result in deterioration of the contractile dysfunction by energy insufficiency.

### 2.5. No Enhancement of Ceramide Levels in CD36KO-STZ Hearts Compared to WT-STZ Hearts

**Figure 5:**
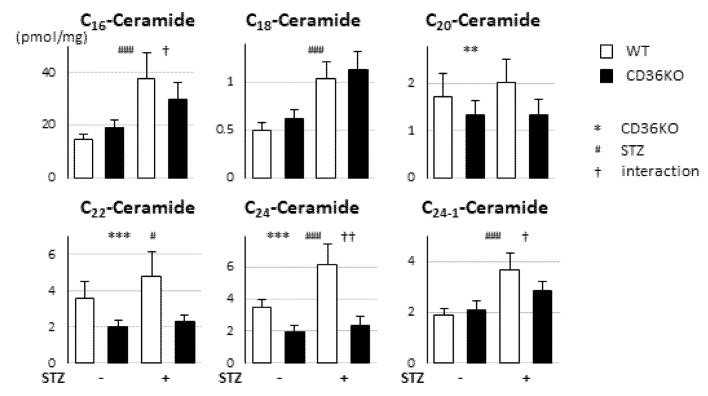
Accumulation of cardiotoxic lipids was not enhanced in CD36KO-STZ hearts compared to WT-STZ ones. Hearts were isolated after a 6 h fast. Ceramides with different lengths of FAs—C_16_, C_18_, C_20_, C_22_, C_24_ and C_24:1_—were measured (*n* = 5–7). ** *p* < 0.01, *** *p* < 0.001; main effect for genotype. ^#^ *p* < 0.05, ^###^ *p* < 0.001; main effect for STZ treatment. ^†^ *p* < 0.05, ^††^ *p* < 0.01; interaction between genotype and STZ treatment.

Diabetes induces an imbalance between FA uptake and oxidation, which leads to the accumulation of cardiotoxic lipids such as ceramides. The amounts of most ceramides with different lengths of FAs (C16, C18, C22, C24 and C24-1) were elevated by STZ treatment, but the elevation levels tended to be lower in CD36KO-STZ hearts than WT-STZ ones (Figure 5). Thus, accumulation of lipotoxic lipids is unlikely to be a major reason for deterioration of contractile dysfunction in CD36KO-STZ hearts.

### 2.6. Synergistic Exacerbation of Contractile Dysfunction by Increased Workload in CD36KO-STZ Hearts

**Figure 6:**
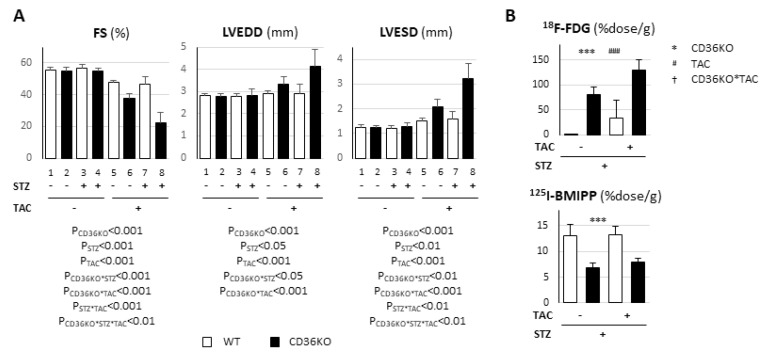
Cardiac contractile dysfunction was synergistically enhanced by STZ and TAC in CD36KO mice. Four weeks after STZ i.p., mice were subjected to TAC. (**A**) Cardiac contractile function was estimated by echocardiography seven days after TAC (*n* = 5–9). (**B**) Uptake of ^18^F-FDG and ^125^I-BMIPP was estimated seven days after TAC (*n* = 6–7). *** *p* < 0.001; main effect for genotype. ^###^ *p* < 0.001; main effect for STZ treatment.

Next, we studied whether the increase in workload due to TAC affected cardiac contraction in CD36KO-STZ mice [9,14]. Four weeks after STZ treatment, mice were subjected to TAC operations. Interestingly, FS was synergistically reduced by TAC and STZ treatment in CD36KO mice (Figure 6A, bar 8), suggesting an accelerated energy shortage against elevated energy demand. Compared to LVEDD, LVESD was more significantly affected by combined stresses in CD36KO mice (Figure 6A, bar 8). In contrast, synergistic exacerbation of contractile dysfunction by TAC was not observed in WT-STZ hearts (Figure 6A, bar 7), which further implicates the importance of FA use in the heart as a primary fuel. A tracer study with ^18^F-FDG and ^125^I-BMIPP revealed that insulin-independent glucose uptake was enhanced by TAC in WT and CD36KO hearts even under STZ treatment, while FA uptake was unaffected (Figure 6B). These findings suggest that energy insufficiency due to STZ treatment becomes more prominent due to the elevation of energy demand by TAC, which could lead to the synergistic exacerbation of contractile dysfunction by STZ and TAC in CD36KO hearts.

## 3. Discussion

It has long been suggested that CD36 is involved in the development of diabetic cardiomyopathy [1,6,11]. Our study exhibited contradictory results, indicating that contractile dysfunction was exacerbated in CD36KO-STZ hearts due to energy insufficiency. FA uptake was markedly reduced in CD36KO hearts at the baseline, which was not changed by STZ treatment. Although glucose uptake was considerably elevated with and without STZ in CD36KO hearts, glycolytic flux into the TCA cycle was largely suppressed by STZ treatment. Consequently, total energy supply, estimated by the pool size in the TCA cycle, was significantly reduced in CD36KO-STZ hearts. The notion that CD36KO-STZ hearts were energetically compromised was further supported by additional mechanical stress by TAC. An increased workload by TAC, which elevates energy demand, synergistically worsened the contractile dysfunction in CD36KO-STZ hearts. A lack of additive increase in myocardial ceramides in CD36KO-STZ hearts suggested that lipotoxicity was not a major reason for contractile dysfunction in this case. Taken together, with regard to energy homeostasis, enhanced FA uptake in diabetic hearts seems to be an adaptation to compensate for a reduced energy supply from glucose. Accordingly, limited FA use from CD36 ablation could contribute to the deterioration of contractile dysfunction in diabetes, rather than to the protection from lipotoxic effects.

### 3.1. Is CD36 a Detrimental Factor for Diabetic Cardiomyopathy In Vivo?

Our data are the opposite to the hitherto known concept that CD36 is a detrimental factor for diabetic cardiomyopathy. We found that there are at least four possible issues that previous studies have overlooked. First and most importantly, the prevailing hypothesis is not supported by in vivo experiments. It has been reported that myocardial CD36 expression/sarcolemmal translocation is increased under diseased conditions such as diabetes and hyperlipidemia [8,15,16,17]. Researchers suggested that enhanced CD36 expression and its relocation to the sarcolemma not only facilitate FA transport and oxidation but also lead to the accumulation of cardiotoxic lipids such as diacylglycerol and ceramides, which, in turn, inhibits insulin signaling and triggers oxidative stress and inflammatory responses [1,6,11]. Indeed, contractile dysfunction was attenuated in CD36 KO mice in special cases of lipid-mediated cardiac dysfunction models [12,13]. However, conclusive in vivo studies indicating a causal link between expression levels/subcellular localization of CD36 and cardiac function in diabetes have never been demonstrated. Second, it is also well-known that PPARα, a master regulator for FA consumption, transactivates CD36 expression [18,19]. Although some studies demonstrated that induction of CD36 is accompanied by induction of PPARα in diabetic hearts, their induction levels vary a lot depending on various factors, including models of diabetes, used animals and the time course [20,21,22,23,24,25]. Therefore, it is still uncertain to what degree the PPARα–CD36 axis is involved in lipotoxic states in diabetic cardiomyopathy. Third, insulin signaling regulates the translocation of CD36 from the endosome to the sarcolemma [1,6,11]. Hyperinsulinemia is suggested to facilitate permanent relocation of CD36 to the sarcolemma, which can cause an oversupply of FA to the cardiomyocytes. However, it is uncertain whether or not type I diabetes (loss of insulin) induces the relocation of CD36 and resultant FA metabolism in the heart. Fourth, CD36 expression is more abundant in the capillary endothelium than in cardiomyocytes [7,8] and capillary endothelial CD36 play a predominant role in FA transport in the heart [7]. It also remains unknown whether subcellular localization of CD36 in the capillary endothelium is affected in diabetes. Thus, there has been no direct in vivo evidence showing that expression levels/subcellular localization of CD36 play a role in the development of diabetic cardiomyopathy. This is the first in vivo study to demonstrate a causal link between CD36 expression and diabetic cardiomyopathy.

### 3.2. Association between Cardiac Metabolism and Contractile Function

We have reported that cardiac contraction is reduced when the energy supply does not meet the energy demand in mice with genetic deletion of genes associated with FA uptake, including CD36KO and fatty acid binding protein 4 (FABP4)/FABP5 double-KO mice [9,14,26]. The concept of association between cardiac contraction and contractile function is summarized below. Despite a significant decrease in the total energy supply, contractile function is preserved in CD36KO and FABP4/5 double-KO mice [9,14,26], because the required energy expenditure is also small at rest [27]. Cardiac contraction is reduced when they are subjected to the following situations: (1) increased workload by TAC and (2) reduced energy supply by STZ-induced diabetes. The heart requires a greater energy supply to maintain the normal contraction against an increased afterload by TAC [27]. However, in CD36KO and FABP4/5 double-KO mice, the compensatory energy use for the increased afterload fails because of a reduction in FA uptake and loss of compensatory increase in the glycolytic flux into the TCA cycle [27]. In the case of STZ-induced diabetes, reduced FA uptake and suppressed glycolysis cause energetically compromised hearts [26,27]. When the energy demand is elevated by TAC in such energetically compromised hearts, the cardiac energetics further deteriorate, leading to severe suppression of cardiac contraction, as shown in this study.

In conclusion, our results indicate that CD36-mediated FA uptake is required for sufficient energy supply to maintain contractile function in STZ-induced diabetes. Enhanced FA use appears to be protective rather than toxic in this model. It is necessary to further study whether the working hypothesis is also applicable to animal models of type II diabetes.

## 4. Materials and Methods

### 4.1. Mice

Mice deficient in *CD36* with C57BL6j background were raised as described elsewhere [28,29]. Mice were housed in a temperature-controlled room with a 12 h light/12 h dark cycle and given unrestricted access to water and standard chow (CE-2, Clea Japan, Tokyo, Japan).

### 4.2. STZ-Induced Diabetic Model, TAC and Sample Preparation

For the type-1 diabetic model, 10–12-week-old male mice were treated with i.p. STZ in 0.1 M sodium citrate (pH 4.5) at a dose of 50 mg/kg for five days [26]. Four weeks after STZ treatment, pressure overload was produced by TAC as described previously [9,14]. Blood was collected from the retro-orbital plexus and then centrifuged at 1500× *g* for 15 min at 4 °C to obtain serum for the measurement of biochemical parameters. After cervical dislocation, the hearts were dissected, snap-frozen in liquid nitrogen and stored at −80 °C until further use. Part of the hearts was fixed with 10% formalin and fibrosis was estimated by Masson’s trichrome stain.

### 4.3. Cardiac Function and Hemodynamic Parameters

The in vivo cardiac function and the heart rate were assessed by transthoracic echocardiography (EUB-7500, Hitachi, Tokyo, Japan) in conscious mice as described previously [9,14]. Blood pressure data were obtained from the average of three-time measurements by the tail cuff method (MK-2000ST, Muromachi Kikai, Tokyo, Japan) [26,30].

### 4.4. Measurement of Blood Metabolites and Glycogen in Hearts

Serum levels of glucose (glutest sensor; Sanwa Kagaku, Aichi, Japan), triglyceride (Triglyceride E-test; Wako Chemical, Osaka, Japan), non-esterified fatty acid (NEFA C-test; Wako Chemical, Osaka, Japan), β-hydroxybutyrate (EnzyChrom Ketone Body Assay Kit; BioAssay Systems, Milpitas, CA, USA), insulin (Mouse Insulin ELISA; Mercodia, Uppsala, Sweden) and lactate (Lactate Colorimetric Assay Kit; BioVision, Milpitas, CA, USA) were measured according to the manufacturers’ protocols [9,14,26]. Glycogen in hearts was measured as described previously [30].

### 4.5. Biodistribution of 125I-BMIPP (15-(p-iodophenyl)-3-(R,S)-methyl pentadecanoic acid) and ^18^F-FDG (2-fluorodeoxyglucose)

The biodistribution of ^125^I-BMIPP and ^18^F-FDG was determined as described previously [31,32]. Mice received intravenous injections of ^125^I-BMIPP (5 kBq) and ^18^F-FDG (100 kBq) via the lateral tail vein in a volume of 100 μL. ^125^I-BMIPP was a gift from Nihon Medi-Physics Co. Ltd. (Tokyo, Japan) and ^18^F-FDG was obtained from batches that were prepared for clinical PET imaging at Gunma University. The animals were sacrificed two hours after injection. The isolated hearts were weighed and counted using a well-type gamma counter (ARC-7001; ALOKA, Tokyo, Japan). Each experiment was performed at least twice.

### 4.6. Metabolome Analysis by Capillary Electrophoresis–Mass Spectrometry

The mice were anesthetized with isoflurane and the ventricles were immediately removed from the mice after a 6 h fast. The heart samples were freeze-clamped using aluminum blocks precooled in liquid nitrogen and maintained at −80 °C. Metabolome analyses were carried out as described elsewhere [9,14,26,30].

### 4.7. Tracing Study with ^13^C_6_-Glucose

Six hours after fasting, ^13^C_6_-glucose (1 mg/g) was intraperitoneally injected into the mice. Ten minutes later, the ventricles were isolated and metabolome analyses were conducted as described previously [9,14,26,30].

### 4.8. Measurement of Ceramides

Ceramide measurement was performed using a triple quadrupole mass spectrometer coupled with a liquid chromatograph (LCMS-8050 system; Shimadzu, Kyoto, Japan) as described previously [26].

### 4.9. Statistical Analyses

The statistical analyses were performed with IBM SPSS (version 24; IBM, Armonk, NY, USA). The data are presented as the mean ± standard deviation. The equality of variance was estimated by Levine’s test. The student’s *t*-test was performed for the two groups’ comparison. A two-way analysis of variance (ANOVA) was used to analyze the effects of genotype (WT vs. CD36KO), STZ treatment (control vs. STZ) and their interaction. * *p* < 0.05, ** *p* < 0.01, *** *p* < 0.001; main effect for genotype. ^#^ *p* < 0.05, ^##^ *p* < 0.01, ^###^ *p* < 0.001; main effect for STZ treatment. ^†^ *p* < 0.05, ^††^ *p* < 0.01, ^†††^ *p* < 0.001; interaction between genotype and STZ treatment. A three-way ANOVA was used to determine whether there were any interaction effects on cardiac function among genotype, STZ treatment and TAC operation. A *p*-value <0.05 was considered to be statistically significant.

## Data Availability

All data and materials are available on request from the corresponding author. The data are not publicly available due to ongoing researches using a part of the data.
